# Personalia

**Published:** 1914-07-25

**Authors:** 


### Personalia.

The Nelson Hospital has received the handsome gift of a piano—the cost defrayed by sums collected by Mr. and Mrs. Risdon Palmer and friends, who have taken a leading part in providing singing for the patients on Sunday evenings.

Mr. Richard Davey Boase, M.R.C.S., L.R.C.P., aged fifty-four, of Penzance, formerly practising at Luxor, Upper Egypt, who died intestate, has left estate valued at £1,639.

